# Anterior Lens Subluxation and Pseudohypopyon

**DOI:** 10.1155/crop/1289814

**Published:** 2026-05-25

**Authors:** Faiza Sarwar, Ahmed Alshaikhsalama, Amer Alsoudi, Ronald Gross

**Affiliations:** ^1^ Department of Ophthalmology, University of Texas Southwestern Medical Center, Dallas, Texas, USA, utsouthwestern.edu; ^2^ Department of Ophthalmology, William Beaumont Hospital, Royal Oak, Michigan, USA, beaumont.edu; ^3^ Department of Ophthalmology, Baylor College of Medicine, Houston, Texas, USA, bcm.edu

**Keywords:** acute angle closure, asteroid hyalosis, lens subluxation, pars plana vitrectomy

## Abstract

Asteroid hyalosis is a benign condition caused by refractile particles in the posterior segment, typically discovered incidentally during eye examination. Rarely, these particles may migrate to the anterior chamber due to lens subluxation, trauma, or pressure changes and mimic a pseudohypopyon or iris metastasis. This case report describes a 67‐year‐old man with acute, painful vision loss in the left eye and elevated intraocular pressure. Slit lamp examination revealed anterior lens subluxation with a pseudohypopyon. This case highlights the diagnostic challenge of asteroid hyalosis presenting as a pseudohypopyon. Thus, a pseudohypopyon in patients with lens subluxation, traumatic lens changes, or pressure gradients should prompt consideration of asteroid hyalosis migration, as delayed diagnosis could impair vision. Early recognition of the potential for migration of asteroid hyalosis, especially in an acute presentation, is crucial for effective management.

## 1. Case Summary

A 67‐year‐old man presented with acute, painful vision loss in the left eye. He reported a remote history of left ophthalmic trauma three decades ago as well as a diagnosis of asteroid hyalosis in both eyes. He also had a history of pseudoexfoliation and lens subluxation without glaucoma, secondary to zonular weakness that moved the lens forward. His medical history was notable for hypertension, hyperlipidemia, and Type 2 diabetes. At baseline, visual acuity was 20/25 in both eyes.

**Figure 1:**
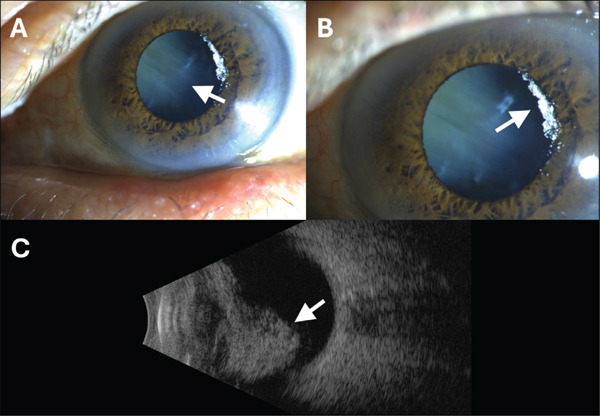
The white arrows reference the pathology indicated in the figure caption, that is, (A) anterior subluxation, (B) anterior migration of asteroid hyalosis , and (C) echogenic opacities.

On exam, his visual acuity was 20/25 in the right eye and 20/400 in the left eye. The intraocular pressure (IOP) was elevated to 12 and 56 in the right and left eyes, respectively. Slit lamp examination revealed anterior lens subluxation (Figure 1A). Anterior segment exam demonstrated a pseudohypopyon in the anterior chamber (Figure 1B). B‐scan ultrasonography revealed echogenic opacities posterior to the lens (Figure 1C). No additional pertinent findings were observed, and no specimens were sent due to low concern for infection.

To address the acute presentation for painful vision loss, initial management included urgent IOP reduction with topical atropine to deepen the anterior chamber and reopen the angle. Two weeks later, surgical intervention was done with a pars plana lensectomy and vitrectomy to relieve the pupillary block. Scleral fixation of the intraocular lens was performed, the hyaloid was lifted, and anterior chamber washout was performed to remove the pseudohypopyon. At 1 week postoperatively, his vision improved to 20/20 and 20/50 in the right and left eyes, respectively. His IOP also returned to normal limits at 7 mmHg in the left eye. At 3 months postoperatively, IOP was 10.

## 2. Discussion

Asteroid hyalosis is a benign condition typically discovered incidentally during an eye examination. It is caused by multiple refractile particles composed of calcium and phospholipids in the posterior segment. Rarely, these particles may migrate to the anterior chamber, mimicking a pseudohypopyon or iris metastasis [1, 2]. There is minimal literature on this presentation due to obstructions, such as lens subluxation, lens removal, or acute increases in IOP [3]. One case was presented intraoperatively [4].

This case was diagnostically challenging because the asteroid hyalosis migration presented as a pseudohypopyon. The asteroid hyalosis appears to have migrated from the posterior segment to the anterior chamber in the setting of lens subluxation. The observed particles in the anterior chamber could have been misleading without the history of asteroid hyalosis, which indicated the cause of the pseudohypopyon. With communication to the anterior segment in the setting of lens subluxation, asteroid bodies that are normally confined to the posterior vitreous may have been displaced toward the anterior segment [5].

The presentation of asteroid hyalosis is classically asymptomatic, and fundus examination may reveal an obscured view and freely moving small yellow‐white opacities consistent with “stars in the night sky” [6]. No treatment is typically necessary in these cases, but the presence of particles in the anterior chamber may suggest underlying pathology that warrants consideration. In our case, the patient′s acute presentation for painful vision loss was caused by a pupillary block secondary to the lens subluxation. Thus, urgency was required in IOP reduction and surgical intervention with a pars plana lensectomy [7]. With such a diagnostically challenging case, careful examination and history are necessary to prevent delay in appropriate diagnosis and treatment. In this case, failure to recognize an asteroid in the anterior chamber may have led to an unnecessary infectious workup with treatment delays.

Another complication of anterior lens subluxation is angle‐closure glaucoma (ACG). The risk for ACG increases with age as zonules weaken and the cataract matures. However, a shallow anterior chamber, iris–lens diaphragm position, hyperopia, medications, and genetic factors have all been implicated [8, 9]. Treatment of anterior lens subluxation causing ACG is both medical and surgical. Recommended medical treatment includes immediate IOP reduction with a systemic agent such as intravenous mannitol and topical IOP‐lowering agents such as dorzolamide, timolol, or brimonidine [10]. Recommended surgical treatment is lens extraction to remove the pupillary block [11]. Laser peripheral iridotomy and anterior chamber paracentesis can also alleviate an acute IOP rise [7]. Correlation with B‐scan findings or ultrasound biomicroscopy may be necessary for additional evaluation [12]. With prompt IOP management and surgical lens removal, patients tend to experience improved visual outcomes.

## 3. Conclusion

This case highlights the possible mechanism underlying asteroid hyalosis migration, which posed a diagnostic challenge in this case due to its presentation as a pseudohypopyon. With such an acute presentation, delays in evaluating and managing the underlying cause could have been detrimental to the patient′s vision. This underscores the importance of understanding the potential for asteroid hyalosis migration, especially in the context of ACG. The presence of a pseudohypopyon in a patient with a history of lens subluxation, traumatic lens changes, or pressure gradient changes is key to considering the migration of asteroid hyalosis. This case emphasizes that early identification of asteroid hyalosis migration can prevent treatment delays in identifying the underlying pathology of an acute presentation.

## Funding

No funding was received for this manuscript.

## Consent

Written informed consent has been obtained to publish the details from the affected individual.

## Conflicts of Interest

The authors declare no conflicts of interest.

## Data Availability

All data is included in this manuscript and supporting documents. Please contact the authors for more information.
